# *In vitro* models of muscle spindles: From traditional methods to 3D bioprinting strategies

**DOI:** 10.1177/20417314251343388

**Published:** 2025-07-23

**Authors:** Yuannan Kang, Deepak M. Kalaskar, Darren J. Player

**Affiliations:** 1 Centre for 3D Models of Health and Disease, Division of Surgery and Interventional Science, Faculty of Medical Sciences, University College London, UK; 2 UCL Division of Surgery and Interventional Science, Royal Free Hospital Campus, University College London, UK

**Keywords:** skeletal muscle, muscle spindle, intrafusal fibre, proprioception, tissue engineering, three-dimensional (3D) bioprinting, bioprinting, bioink, hydrogel

## Abstract

Muscle spindles are key proprioceptive mechanoreceptors composed of intrafusal fibres that regulate kinaesthetic sensations and reflex actions. Traumatic injuries and neuromuscular diseases can severely impair the proprioceptive feedback, yet the regenerative potential and cell-matrix interactions of muscle spindles remain poorly understood. There is a pressing need for robust tissue-engineered models to study spindle development, function and regeneration. Traditional approaches, while insightful, often lack physiological relevance and scalability. Three-dimensional (3D) bioprinting offers a promising approach to fabricate biomimetic, scalable, and animal-free muscle spindle constructs with controlled cellular architecture. Various bioprinting techniques – including inkjet, extrusion, digital light projection and laser-assisted bioprinting – have been explored for skeletal muscle fabrication, but replicating intrafusal fibre complexity remains a challenge. A major challenge lies in bioink development, where biocompatibility, printability and mechanical strength must be balanced to support intrafusal fibre differentiation and proprioceptive function. Recent molecular insights into spindle anatomy, innervation and extracellular matrix composition are shaping biofabrication strategies. This review discusses the current state of muscle spindle modelling, the application of 3D bioprinting in intrafusal fibre engineering, key challenges and future directions.

## Introduction

Proprioception is a highly coordinated mechanosensitive feedback system that senses the mechanical and spatial status of the muscles, it is vital for the maintenance of normal gait, fine motor skills, posture and coordinated movements in vertebrates. The integration of proprioceptive information to the central nervous system (CNS) contributes to the protective reflex of the body, allowing the body to make fast, involuntary adjustments to maintain stability and reduce the risk of injury.^1^ The principal proprioceptive mechanoreceptor, muscle spindles, are embedded in skeletal muscles and are integral to the stretch reflex arc, giving rise to kinaesthetic sensations and unconscious spinal reflex actions.^2^

These mechanosensory organs are frequently affected in many neurodegenerative and neuromuscular diseases, resulting in altered muscle spindle morphology and loss of proprioceptive feedback. Patients who suffer proprioceptive dysfunction often display poor posture and movement control, balance problems, abnormal tendon reflexes, gait disturbance and motor deficits such as ataxia and dysmetria.^2–6^ These impairments are also considered a secondary effect in diseases such as Parkinson’s disease (PD), diabetic neuropathy, Huntington’s disease (HD) and multiple sclerosis.^7–9^

Muscle spindles are composed of bundles of intrafusal fibres, which originate from the same pool of myogenic progenitors (i.e. satellite myoblasts) as extrafusal muscle fibres.^10,11^ However, they follow distinct differentiation and specialisation pathways, resulting in the formation of two functionally distinct components within skeletal muscle. The mean density, distribution and morphology of muscle spindles are among the many factors governing proprioceptive awareness in skeletal muscles. Significant progress in the molecular biology of the muscle spindle has only emerged in recent years, such as the identification of new markers for intrafusal fibres, capsule cells, sensory and ECM components. Such evidence has begun to unravel the intricate processes underlying muscle spindle development and function.^12–17^

However, the full understanding of the signalling pathways and the transcriptional programmes specific to intrafusal fibres, are yet to be elucidated. Furthermore, the contributions of capsule cells to mechanosensation, the specific interactions between sensory neurons, intrafusal fibres and ECM proteins during spindle formation and maintenance are still not well characterised. Additionally, the regenerative potential of muscle spindles after injury or in disease conditions is largely unknown. Although skeletal muscle displays excellent regenerative capacity, conditions such as volumetric muscle loss (VML), peripheral neuropathies and several degenerative and congenital diseases^18–20^ can lead to significant loss of function. Importantly, in comparison to extrafusal fibres, the regenerative potential of muscle spindles is less understood.^21^

Much of the research focus in recent years, has been on the repair and regeneration of extrafusal skeletal muscle fibres. Given the significance of muscle spindles in proprioceptive function, focusing solely on extrafusal fibre regeneration is insufficient for the optimal repair of skeletal muscles. Due to the huge demand for organ transplantation and donors worldwide, there is an increasing demand for more robust tissue engineered models of the muscle spindle. Such models will aid in the development of transplantable muscle grafts, personalised medicine and pharmacological therapies.^20^ Fabricating de novo muscle spindles offers solutions to the issues of autologous and allogeneic grafts and key challenges in muscle regenerative medicine research, such as insufficient regenerative capacity, scar tissue formation, limited vascularisation and functional restoration.^22^ More importantly, up-to-date models of muscle spindles are required for studies of intrafusal fibre development in vitro, cell-matrix and cell-biomaterial interactions.

Three-dimensional (3D) bioprinting is an emerging technology that could revolutionise skeletal muscle spindle tissue engineering and regenerative medicine by providing scalable, animal-free models, addressing translational and ethical concerns. Recent advances in 3D bioprinting have enabled the automated and high-throughput fabrication of defined 3D tissue constructs using computer-aided design (CAD) models.^20,23,24^ It brings together 3D printing (biomaterials) and bioprinting (cellular printing) technologies to recapitulate the complex structural and biological components of native tissues and organoids with high resolution. These approaches hold the potential to produce biomimetic scaffolds with controlled arrangement of cells and growth factors (GFs), that better mimic their in vivo environment.^23,25^ The layer-by-layer deposition of biomaterials also creates exceptional morphological detail that could not be replicated using other manufacturing approaches.^22,25,26^ By incorporating desired factors, different cell types or patient-derived cells, 3D bioprinting could further accelerate the development of personalised muscle transplantation by manufacturing recipient-specific substitutes,^27^ and also paves the way to tailored disease modelling and drug screening in vitro.^28,29^

This review is devoted to muscle spindles, with a focus on engineering intrafusal fibres using 3D bioprinting technologies. The role of 3D bioprinting in facilitating intrafusal fibre development in vitro is first described, with a comparison to traditional tissue engineering methods. This is followed by an overview of the latest 3D bioprinting techniques for muscle fibre engineering, including a detailed evaluation of various bioprinting strategies and biomaterials. The review then delves into recent advances in understanding the molecular events and pathophysiology of muscle spindle, highlighting how these insights can aid in the design of 3D bioprinting strategies. Key design criteria for intrafusal fibre bioprinting will also be discussed. Finally, the review concludes with a discussion on the current challenges and future perspectives in the field.

## The role of 3D bioprinting in generating muscle spindles in vitro

Over the last decade, tissue engineering (TE) has received increasing attention in regenerative medicine and drug development, as it allows for the generation of human-relevant models in vitro. While tissue engineered scaffolds promise greater functional restoration potential than biomaterials-based and 2D monolayered systems,^30–32^ traditional scaffold fabrication strategies often lack the spatial architecture of native tissue and have poor control over the microgeometries, cell distribution and the delivery of biologically active molecules (Table 1).

**Table 1.** Comparison between traditional TE and 3D bioprinting approaches.

Criteria	Traditional tissue engineering	3D Bioprinting
Biocompatibility	High	Moderate to high; potential cytotoxicity
Mechanical strength	Lower	Higher
Morphological details	Difficult to achieve	Defined morphology; using CAD
Cell distribution	Uneven	Automated; precise placement of cells
Precision	Low; lacks spatial control	High; allows highly organised structures
Complexity	Limited; simple tissue	Capable of creating complex structures
Scalability	Easier for simple constructs	Challenging to scale for larger tissues or full organs
Customisation	Limited	Allows patient-specific scaffolds
Vascularisation	Difficult to achieve	Allow vascularisation
Material flexibility	Wide range of biomaterials available	Limited by printable materials
Cost	Low to moderate	Higher cost due to specialised equipment
Cell integration	Manual cell seeding	Possible for multi-cell type integration
Fabrication time	Slower	Faster
Accessibility	Well-established	Technical complexity

Traditional TE approaches aim to strike a balance between maintaining shape fidelity and stiffness of scaffolds, while optimising cytocompatibility for effective cell spreading and growth. For example, increasing the polymer crosslink density would increase the stiffness of the hydrogel, resulting in greater shape fidelity. However, cells often favour softer and a more aqueous microenvironment as it supports cell migration, cell-matrix, and cell-material interactions.^33,34^ Therefore, most engineered muscle tissues demonstrate moderate shape fidelity, cytocompatibility and compromised mechanical properties, leading to simplified extra-cellular matrix (ECM) microenvironment and intrinsic architecture.

It is also important to note that muscle fibres function via unidirectional forces. To achieve the highly aligned fibre structure in vitro, attempts have been made to promote the parallel orientation. Kroehne et al.^35^ developed a supporting scaffold of collagen sponges using freeze-dry techniques and supported C2C12 muscle cell growth and myotube alignment, both in vitro and in grafts. The collagen I-based sponge consists of parallel pores created by ice crystals formed during freezing, and produced a pore size range from 20 to 50 µm, which was found to promote myotube alignment. The increased level of sarcomeric myosin staining and laminin deposition also suggested that the cells developed into more mature stages, where they were capable of forming their own ECM. Additionally, in transplanted collagen sponge scaffolds, functional restoration was reported as the regenerating muscle fibres produce contractile forces. However, the system only replicates certain aspects of the native tissue, with limited control over internal geometry and cell distribution. In the following section, previous studies on intrafusal fibre modelling using traditional cell and tissue engineering approaches are summarised, followed by a review on the emerging 3D bioprinting strategies for muscle tissue engineering.

### Traditional cell and TE approaches for intrafusal fibres

An unified objective of in vitro studies has been to replicate the in vivo anatomy and characteristics of intrafusal fibres. Several studies using traditional TE approaches have induced differentiated and functional intrafusal fibres in vitro, and different strategies and cell sources have been proposed to recreate the native muscle spindle microenvironment. However, achieving the native spindle structure while controlling the complexity of the culture system has proven to be challenging. In order to identify the critical factors governing intrafusal fibre development and physiology, it is important to consider adopting a minimalist approach when introducing undefined biological components, scaffold design and/or cell types such as nerve and vascular cells. A highly controlled system would facilitate the investigation of the underlying mechanisms that regulate intrafusal fibre development and functions by altering individual factors.

N-1[3-(trimethoxysilyl)propyl]-diethylenetriamine (DETA)-based fabrication technique is a serum-free and non-biological culture system, and have been used to generate skeletal muscles in vitro. Multiple cell sources have been cultured on DETA-coated surfaces by previous studies, resulting in intrafusal fibre specification and functional maturation. Rumsey et al.^36^ demonstrated a dose-dependent effect of neuregulin isoform 1-β-1 (NRG 1-β-1) in promoting nuclear bag phenotype in intrafusal fibres using embryonic rat muscle cells. An 81% increase in nuclear bag formation was observed when the NRG 1-β-1 concentration increased from 10 to 100 ng/ml, along with the co-expression of phosphorylated ErbB2 and Egr3 in BA-G5 positive nuclear bag fibres, confirming the role of NRG-1/ErbB2/Egr3 signalling pathway in the formation of intrafusal fibres and the specific role of the transcription factor, Egr3, in intrafusal fibre development. All myotubes cultivated on DETA-coated coverslips exhibited distinct nuclear bag morphology and positive expressions of alpha cardiac-like MHC, providing insights into the minimal components necessary for intrafusal fibre specification. Electrophysiological properties of isolated nuclear bag fibres were also characterised, further indicating the establishment of a functional system derived from serum-free formulation under controlled conditions.

Human skeletal muscle stem cells (hSKM SCs) have also shown the capacity to differentiate into intrafusal fibres on DETA surfaces, they are ideal progenitors for the investigation of muscle repair and regeneration as they are highly functional, self-regenerative and easy to isolate. In particular, the myogenic potential of satellite cells governs the extraordinary regenerative and differentiation capacity of skeletal muscles. Guo et al.^37^ demonstrated Egr3 and s46-positive myotubes following immunocytochemical analysis of hSKM SCs on DETA surfaces, further supporting previous findings.^36,38–40^ Moreover, various components of the neuromuscular reflex arc have been incorporated into the in vitro model of intrafusal fibres and demonstrated neuronal growth using DETA-based system.^37,41,42^ Functional synaptic connections can be established when co-culturing hSKM SC-derived intrafusal fibres with human sensory neurons, as indicated by the flower spray endings around intrafusal fibres with positive PRKCA-binding protein (PICK1).^37^ In addition to the NRG-1 treatment, the serum-free medium was supplemented with laminin and agrin during the differentiation phase of myotubes, facilitating the induction of intrafusal fibres via NRG-1/ErbB2/Egr3 signalling pathway. As a result, a five-fold increase in the formation of nuclear bag fibres was reported in comparison to the control treated with NRG-1 only.^37^

Embryonic murine myocytes and DRG sensory neurons have been co-cultured to evaluate the mechanosensory system in vitro.^42^ NBActiv4 medium was used to support both type Ia and type II sensory innervations, with an 11% and 10% increase in the number of annulospiral (ASW) and flower-spray endings (FSW) by the end of the experiment (Day 23). Field stimulation of intrafusal fibre led to a spatiotemporal influx of Ca^2+^ along the primary sensory neuron axon, as examined using Fluo-4 AM dye, indicating functional afferent activity in the co-culture system. In contrast, cultures with only DRG neurons showed no subsequent Ca^2+^ flux. Furthermore, the colocalisation of functional and structural proteins, brain sodium channels 1 (BNaC1) and protein kinase C alpha (PRKCA1 or PICK1), were found at both terminals of sensory afferents, supporting the idea that functional connections between Ia afferents and intrafusal fibres can be established in vitro.^42^

In comparison, human induced pluripotent stem cells (iPSCs) allow for the generation of patient-specific cells and avoid ethical concerns associated with embryonic stem cells. iPSCs are obtained from human donors and generated by reprogramming adult somatic cells back to pluripotency through the introduction of defined transcription factors, thus acquiring the ability to differentiate into any cell lineage.^43^ Recently, Colón et al.^44^ integrated human iPSC-derived myoblasts into a DETA-based body-on-a-chip system and generated intrafusal-specific nuclear bag and nuclear chain fibres following NRG treatment, similar to that observed in intrafusal fibres generated from human satellite cells^37,38^ and in vivo studies.^45^ The immunocytochemical analyses revealed a three-fold increase in the number of intrafusal fibres with positive S46 and pErbB2 staining, indicating the key regulatory role of NRG in the formation of intrafusal fibres across multiple muscle progenitor populations. This also underscores the plasticity of muscle progenitor cells in expressing intrafusal fibre-specific MyHC markers, under appropriate signalling conditions. The use of iPSCs also improves the clinical relevance of the model as disease-specific iPSCs could be used for personalised medicine, disease modelling and drug screening. Previous studies also demonstrated the efficiency of iPSCs in generating neuromuscular reflex arc components including extrafusal muscle fibres, functional sensory and motor neurons.^46,47^ Therefore, iPSC-derived intrafusal fibres can now be integrated for the in vitro modelling of patient-specific conditions such as muscular dystrophy, volumetric muscle loss, sarcopenia, and neuromuscular diseases.

Although using primary human tissues in research offers significant benefits because of their great physiological relevance and genetic authenticity, it comes with several limitations that impact the reproducibility and feasibility of experiments. Strict regulations and ethical concerns have been applied to protect the rights of donors, the validity of scientific research and avoid the exploitation of donors, which may lead to a limited supply of human tissue and expensive procurement. Due to the biological and physiological characteristics that exist between tissue or cell samples obtained from different donors, the reproducibility of experiments and data interpretation could also be significantly influenced. Researchers have limited control over individual variations such as the genetic profile, health status, sex, age and epigenetic factors, which can contribute to significant variability in vitro. Also, the maintenance of primary human tissue and cells requires careful handling and processing, require high-cost reagents (e.g. specialised culture media, growth factors, coatings such as collagen and Matrigel, etc.), have limited growth and functional capacity and also pose technical challenges.

Myogenic cell lines are therefore valuable tools for in vitro modelling of skeletal muscle, offering high reproducibility, cost efficiency and sustainability. The C2C12 cell line is a well-documented, commonly used murine myoblast cell line for exploring myogenesis, muscle physiology and regeneration. It is an immortalised sub-clone of mouse myoblasts originally isolated by Yaffe and Saxel,^48^ which they cultured from 2-month-old C3H mice thigh muscle 70 h after crush injury. Due to their high accessibility, C2C12 myoblast-derived skeletal muscle models have been widely adopted in laboratories and utilised in both two-dimensional (2D) and three-dimensional (3D) systems.^49,50^ It is affordable and simple to maintain, demonstrating high fusion rate and differentiation with the ability to form force-generating contractile muscle fibres.

Previous work from our laboratory^49^ established a minimalistic model of intrafusal-like myotubes using C2C12 myoblasts. The effect of NRG-1 supplementation on C2C12 myotubes was investigated at morphological, molecular, and transcriptional levels. The diameter difference ratio (DDR) was calculated to define the structure of nuclear bag fibres (myotubes) and nuclear chain fibres within a heterogeneous population, which revealed that nuclear bag myotubes exhibited a mean DDR ratio twice as high as that of linear myotubes. Following 100 ng/ml of NRG-1 supplementation, fluorescent microscopy showed an approximately 80% increase in the number of nuclear bag myotubes, corroborating previous findings.^36–38,44^ Additionally, while the expression of Egr3 was significantly elevated and paired with the development of nuclear bag myotubes, it was also present in the control population, indicating that the specific development of intrafusal fibres is driven by the NRG-1/ErbB2/Egr3 signalling pathway. The expression of MyHCs was investigated for an in-depth characterisation of the model. However, the only increase following NRG-1 treatment was observed in Myh4, suggesting a shift towards a more contractile and mature phenotype, rather than intrafusal-specific differentiation. Meanwhile, MyHC 3, 6 and 8 were downregulated, and no immunoreactivity was detected for s46, which contrasts with previous literature.^36–39,44^ However, significant increases in the level of Myod1, Etv4 and PAX7 were reported, highlighting an enhanced proliferation phase in the NRG-1 treated satellite cells and fusion potential during the differentiation process.

Despite several attempts to replicate the intrafusal-specific morphology, protein profiles and MyHC profiles in vitro (see Table 2), a comprehensive characterisation of intrafusal fibres has not been achieved using traditional methods. This may be attributed to the limited architectural complexity and shortened culture periods in traditional cell and TE systems, resulting in reduced degree of myotube alignment and functionality. Maintaining myotubes on culture dishes for extended periods is also challenging due to their contractile properties. As myotubes develop and mature, they generate mechanical tension, often leading to detachment from the surface, particularly in monolayer muscle cell cultures. In addition, only certain aspects of muscle spindle behaviour have been addressed in most models. There is a technical limitation that hinders the production of larger-scale models and investigations into factors such as the impact of microarchitecture on myotube differentiation, functional integration with other cell types, and the physiological relevance of the model. More importantly, most studies often rely on a limited set of markers due to the restricted understanding of muscle spindles at the time. Previous findings have provided valuable initial insights but were constrained by the available tools and markers. As our understanding of intrafusal fibre and muscle spindle biology has expanded and new markers have been identified, more comprehensive approaches can now be employed to better define and characterise intrafusal fibres in greater detail.

**Table 2.** Cell and TE methods for generating intrafusal fibres in vitro.

Cells	Culture	Biomaterial	Supplementation	Expression	Results	Ref.
Human iPSCs	2D	DETA-modified and collagen coated glass coverslips	hEGF, insulin, NRG, agrin, laminin, GDNF, BDNF, CNTF, NT3, NT4, vitronectin, IGF-1, cAMP, Shh, RA, NGF	pErbB2, slow MHC	- Cell viability (high) - Alignment (high) - Culture period (20 days) - Myotube differentiation (high)	Colón et al.^44^
Human satellite cells + human sensory neurons	2D	DETA-modified and collagen coated glass coverslips	Insulin, EGF, Gentamicin, NRG, laminin, agrin	Slow tonic MHC, alpha cardiac-like MHC, pErbB2, Egr3, pheripherin, PICK1	- Cell viability (high) - Alignment (high) - Culture period (14 days) - Myotube differentiation (high) - Functional innervation (high)	Guo et al.^37^
Human satellite cells + human gamma-motoneurons	2D	DETA-modified and collagen coated glass coverslips	Insulin, EGF, Gentamicin, NRG, laminin, agrin	pERB2, Egr3, ERR-gamma	- Cell viability (high) - Alignment (high) - Culture period (18 days) - Myotube differentiation (high) - Functional innervation (high)	Colón et al.^38^
C2C12	2D	N/A	NRG-1	All MHC isoforms except from decreased expression of MHC 3, 6 8; Egr3, ETV4, MyoD1, PAX7	- Cell viability (high) - Alignment (high) - Culture period (11 days) - Myotube differentiation (high)	Barrett et al.^49^
Human primary myoblast, C2C12	2D	N/A	NRG, agrin	Egr1, Egr2, Egr3, RAB6, sarcosine, kinesin family member 5B, slow MyHC, neonatal MyHC	- Cell viability (high) - Culture period (2 days) - Myotube differentiation (high)	Jacobson et al.^39^
Rat primary myoblasts + DRG explants	2D	N/A	NRG-1β	GAP-43, TrkC, NF-200	- Cell viability (high) - Myotube differentiation (high)	Qiao et al.^40^

### 3D bioprinted skeletal muscle constructs

To address the difficulties in reproducing biomimetic tissue constructs, such as lack of structural complexity and the heterogeneous cell distribution, progressing towards 3D skeletal muscle bioprinting provides a suitable technology. 3D bioprinting automates most of the fabrication processes, allowing for greater precision, functional complexity, scalability and customisation.^51^ The complex structure and components of the native tissue must be considered in engineered constructs, as inadequate control over spatial organisation and bioactive molecules greatly limits the ability to recreate biomimetic complex structures.

3D bioprinting approach offers more precise patterning of the cells and bioinks, as well as the capability to incorporate multiple cell types, biomaterials and delivery system that closely mimics the native microenvironment, where a wide variety of ECM materials, cell types and biomolecules are found.^20,24,52–54^ It not only allows for the production high-quality skeletal muscle constructs, but also enables the customisation of complex and large-scale tissue construct with consistency.^54,55^ This aspect is crucial for future clinical applications, where large volumes of muscle tissue might be required. The ability to fine tune the mechanical and biochemical properties of the bioinks, also ensures that the engineered tissue closely mimics the natural mechanical environment of muscle, which potentially leads to better muscle regeneration and functional integration with host tissues.^24,54^ Additionally, these advancements are crucial for developing effective models to predict human physiological responses in vitro, or artificial grafts for transplantation that could potentially reverse muscle spindle diseases and degenerative conditions, such as VML, peripheral nerve injury and muscular dystrophies.

For skeletal muscles, generating extrafusal fibres in vitro have always received more attention than intrafusal fibres, due to their higher clinical relevance and the lack of understanding of muscle spindle biology. Table 3 summarises novel methods for bioprinting aligned and contractile myotubes, where various bioprinting strategies, biomaterials and cell sources have been employed. These approaches demonstrate greater cellular alignment, viability, maturity and contractility than traditional engineered muscles that were encapsulated in bulk matrices.^56–62^ Promising results with functional restoration have also been observed following in vivo implantation up to 2 weeks.^58^

**Table 3.** 3D bioprinting approach for muscle engineering.

Biomaterials	Cells	Bioprinting technique	Scaffold design	Marker expression	Mechanical properties	Results	Ref.
Gelatin + Fibrinogen + HA + Glycerol	C2C12	Extrusion printing	- 15 mm × 15 mm × 1 mm - Porous structure surrounded by outer PCL framework - Fibre diameter = 250 µm	MHC, desmin, a-BTX, vWF	/	- Cell viability (high) - Alignment (high) - Culture period (2 weeks) - Myotube differentiation (high) - Myotube regeneration (high) - Force generation (high)	Kang et al.^58^
PEG-Fibrinogen + alginate	C2C12	Extrusion printing (co-axial)	- 7-layered aligned rectilinear construct (120 µm × 50 µm) - Fibre diameter = 250 µm	MyHC, ACTA1, MYH2, MyoG, MyoD1, laminin	Young’s modulus = 48 kPa (prior to EDTA wash)	- Cell viability (high) - Alignment (high) - Culture period (21 days) - Myotube differentiation (high but with a small delay)	Costantini et al.^63^
Fibrinogen + gelatin + HA + glycerol + PCL	hMPCs + hNSCs	Extrusion printing (customised)	- PCL anchors printed in each layers of printed cell-laden bundle (10 mm × 7 mm × 3 mm) - Fibre diameter = 300 µm	NF, MHC, GFAP, AChR	/	- Cell viability (high) - Alignment (high) - Culture period (8 weeks) - Myotube differentiation (high) - Myotube regeneration (high) - Force generation (high)	Kim et al.^64^
PEG-Fibrinogen	Mabs/ hMSCs	Extrusion printing	- Parallel fibre extruded on a rotating C-shaped support - Fibre diameter = 300 µm	Laminin, desmin, MyHC, SMA, vWF, pNF, PAX7, a-BTX	/	- Cell viability (high) - Alignment (high) - Myotube differentiation (high) - Myotube distribution (improved) - Myotube regeneration (full) - Force generation (improved)	Fornetti et al.^65^
Decellularized ECM	C2C12	Extrusion printing	- Fibres extruded in parallel, diamond and chain patterns - Supported by PCL framework at both ends of the construct	MHC, agrin, BTX, Myf5, MyoG, MyoD	- Ultimate tensile stress (increased from ~2 kPa on Day 7 to ~3.5 kPa by Day 14) - Elastic modulus (increased from ~9 kPa on Day 7 to ~12 kPa by Day 14)	- Cell viability (high) - Alignment (high) - Culture period (2 weeks) - Myotube differentiation (high)	Choi et al.^57^
Alginate + pluronic PF127	C2C12	Extrusion printing	- 4-layered parallel-aligned squared construct - Fibre diameter = 250 µm	MyoG, ACTA1, MyoD	/	- Cell viability (high) - Alignment (high) - Culture period (7 days) - Myotube differentiation (high)	Mozetic et al.^66^
Alginate + gelatin	Mouse primary cells	Extrusion printing	- 10 mm × 10 mm with 1 mm spacing - Fibre diameter = 200 µm	/	- Printability (improved)	- Cell viability (high) - Culture period (2 days)	Chung et al.^67^
GelMA + alginate methacrylic	C2C12	Extrusion Printing	- A 2-layered circle containing aligned filaments - Fibre diameter = 200 µm	F-actin, MHC	Young’s modulus = 5.53 ± 2.01 kPa	- Cell viability (high) - Alignment (high) - Culture period (2 weeks) - Myotube differentiation (high)	García-Lizarribar et al.^68^
GelMA + alginate	C2C12	Extrusion printing	- /	F-actin, desmin	- Compressive modulus (increased) - Viscosity (increased) - Storage and loss moduli (increased)	- Cell viability (high) - Alignment (high) - Culture period (12 days) - Myotube differentiation (high) - Myotube metabolic activity (increased)	Seyedmahmoud et al.^69^
Oxidised alginate + gelatin	C2C12	Extrusion printing	- 3-layered parallel-aligned squared construct - Fibre diameter = 250–330 µm	/	/	- Cell viability (high) - Alignment (high) - Myotube differentiation (high)	Distler et al.^70^
Alginate + GelMA + HEC	C2C12	Extrusion Printing	- Fibre diameter = 100–200 µm - Inner channel diameters = 22–85 µm	MyoD, MyoG, ACTA1	/	- Cell viability (high) - Alignment (high) - Culture period (7 days) - Myotube differentiation (high)	Bolívar-Monsalve et al.^71^
GelMA + AuNPs/ MXene nanosheets	C2C12	Extrusion printing	- Dot shape - Fibre diameter = 200 µm	MHC	rheological properties (improved)	- Cell viability (high) - Alignment (high) - Myotube differentiation (high)	Boularaoui et al.^72^
GelMA + collagen + decellularized ECM	C2C12, hASC	Customised extrusion printing (photocrosslinked within the nozzle)	- Mesh structure - Fibre diameter = 700 µm	F-actin, MHC, troponin T	Young’s modulus~ 0.7 kPa	- Cell viability (high) - Alignment (high) - Culture period (3 weeks) - Myotube differentiation (high) - Myotube regeneration (high)	Lee et al.^62^
CELLINK^®^ GelXA FIBRIN (Xantan gum + fibrinogen)	C2C12	Extrusion printing	- 1-layered line (20 mm) - Fibre diameter = 350 µm	MyoD, MCK	/	- Cell viability (high) - Culture period (21 days) - Myotube differentiation (low)	Ronzoni et al.^61^
CELLINK^®^ GelMA A (GelMA + alginate)	C2C12	Extrusion printing	- 1-layered line (20 mm) - Fibre diameter = 350 µm	/	/	- Cell viability (moderate to high) - Culture period (14 days) - Myotube differentiation (low)	Ronzoni et al.^61^
CELLINK^®^ FIBRIN (NFC/alginate + fibrinogen)	C2C12	Extrusion printing	- 1-layered line (20 mm) - Fibre diameter = 350 µm	MyoD, MCK	/	- Cell viability (high) - Alignment (high) - Culture period (28 days) - Myotube differentiation (high)	Ronzoni et al.^61^

PF: PEG-FIbrinogen; Mabs: mouse mesoangioblasts; SMA: smooth muscle actin; vWF: von Willebrand factor; pNF: phospho-neurofilament; alpha-BTX: alpha-bungarotoxin; GelMA: Gelatin methacryloyl; hMPCs: human muscle progenitor cells; hNSCs: human neural stem cells; HA: hyaluronic acid; NF: neurofilaments; GFAP: glial fibrillary acidic protein; MyoG: myogenin; ACTA1: sarcomeric actin; AuNPs: gold nanoparticles; MXene nanosheets: transition metal carbide; MHC: myosin heavy chain; hASC: human adipose stem cell; /: not mentioned.

In addition, advanced tissues have been engineered, showcasing the complexity and potential of 3D bioprinting. For example, 3D bioprinted osteochondral (OC) grafts comprising bone marrow stroll cells (BMSCs) have shown superior regenerative capacity and support functional restoration of cartilage-subchondral bones in OC-deficient rats.^73^ Vasculature bioprinting using 3D coaxial nozzles have also been established to generate perfusable vessel-like networks, where microchannels are induced by co-extruding the permanent hydrogel with a sacrificial bioink that could be removed to leave empty channels. This promotes the transportation of nutrients and gases within the bioprinted tissues, thereby improving cell viability over prolonged culture period and allows the formation of thicker vascularised tissues.^71,74–78^ Schwann cell and MSCs have also been incorporated using extrusion-based bioprinting to recapitulate nerve conduits in vitro, where extensive axonal regeneration was observed following implanting the nerve graft in a sciatic nerve injury model in rats.^79^

Mozetic et al.^66^ fabricated layers of parallel-aligned myotubes through 3D bioprinting and reported high cell viability, myoblast differentiation and improved expression of myogenic genes including MyoG, ACTA1 and MyoD, in comparison to monolayer cultures. Pluronic F-127 (PF127) polymers were added into alginate solutions, improving the shape retention of the bioprinted constructs and facilitating the elongation of C2C12 myotubes along the deposition direction. Christensen et al.^80^ recapitulated the orientation and alignment of native myotubes using the assembled cell-decorated collagen (AC-DC) bioprinting design. Cell-laden HA hydrogels were uniformly extruded and coated on pre-defined collagen microfibres, producing aligned 3D fibres as the stepper motor drove the rotation of collagen microfibres to create parallel wrappings. The implantation of AC-DC constructs in rodent VML model significantly enhanced regeneration of the injured area, as demonstrated by an increase in both the number and size of myofibres, resulting in 76% functional recovery. This supports previous findings on the importance of stable structure in guiding the orientation and alignment of 3D bioprinted muscle cells.

Fornetti et al.^65^ demonstrated the beneficial effect of 3D bioprinting in creating more coherent myofibre cultures using PEG-Fibrinogen (PF) hydrogels, in comparison with a cellularised bulk construct. A rotating C-shaped support was connected to an extrusion-based 3D bioprinter, which act as anchor points and provided uniaxial signals to promote the alignment and elongation of myotubes. It was shown that the cellular density, myotubes width and length were significantly higher in bioprinted constructs. The bioprinted constructs also exhibited greater circularity and decreased cross-sectional area, suggesting a more homogeneous myotube distribution and improved maturation compared to myotubes that were randomly oriented in bulk hydrogels. Lee et al.^62^ proposed new photocrosslinking technique utilising a customised extrusion bioprinter, where GelMA-based bioinks were crosslinked within the extrusion nozzle via an integrated optical fibre. By providing a consistent UV light source to the flowing bioink, this method ensured effective crosslinking of each extruded filament with minimal regional variability. This approach enhanced the mechanical strength of multilayered muscle constructs and improved the print fidelity of low-viscosity bioinks. To evaluate its potential, C2C12 myoblast cells were printed through a nozzle featuring microgroove patterning. This setup resulted in high myotube alignment and robust myogenic activity in vitro. Additionally, in vivo experiments were conducted to investigate the regenerative efficiency of human adipose stem cells (hASCs) in a VML model. The results showed a significant increase in HLA-A positive myofibers and improved myotube regeneration compared to constructs printed using conventional bioprinting methods, demonstrating the efficacy of this technique in muscle tissue engineering.

Moreover, recent advancements in 3D bioprinting have focused on fabricating thicker tissues, where the development of efficient transport networks is crucial for nutrient and oxygen exchange to support long-term cell survival and growth. To achieve multi-channel constructs, sacrificial materials are used to create hollow structures within thick constructs, a feat that traditional biofabrication techniques cannot accomplish. It has been shown that cell-laden hydrogels with depths exceeding 200 µm can lead to cellular hypoxia, particularly at higher cell densities.^81^ Kang et al.^58^ developed a porous scaffold using PCL pillars and sacrificial PF-127 to provide structural support and minimise external forces.

This approach was believed to enhance the elastic moduli of the hydrogels, as demonstrated by Daly et al.^82^ Within the PCL framework, C2C12 myoblasts were extruded in a hydrogel mixture of gelatin, fibrinogen, HA and DMEM supplemented with glycerol, forming fibre-like bundles with high cell viability (~97 ± 6%) on day 1 and longitudinal growth from day 3. Myoblasts printed without the PCL frame displayed poor cell alignment with randomly oriented morphologies. Following 7 days of differentiation, the muscle constructs were implanted into ~14-week-old rats, showing functional innervation and vascularisation after 2 weeks, electromyography also confirmed the capacity of the bioprinted construct to generate action potentials within 4 weeks. More recently, Bolívar-Monsalve et al.^71^ investigated the impact of empty channels within bioprinted constructs on muscle cell viability, migration and proliferation. Using a customised mixing printhead, they co-extruded GelMA-alginate hydrogels with hydroxyethyl cellulose as the sacrificial material. PF-127 was not selected as the sacrificial material due to its non-Newtonian behaviour, which could cause clogging during printing and affect the overall resolution of the bioprinted structure. The presence of Ki67 and reduced expression of hypoxia marker HIF1-alpha suggests that hollow microchannels promote higher cell viability, increased metabolic activity and improved myotube alignment along the direction of the channels, compared to bulk hydrogel controls. This effect is largely attributed to the more efficient mass transport within the construct.

Compared to conventional methods, 3D bioprinted muscle constructs promote superior structural fidelity, better cell alignment, and more homogeneous cell distribution. Advances such as multi-material bioprinting, co-printing of multiple cell types and integration with vascular and neural components have enabled the fabrication of more physiologically complex constructs. Together, these advancements provide a robust foundation for adapting bioprinting strategies to more specialised and intricate structures, such as the muscle spindle. The following section reviews the major 3D bioprinting techniques and bioink formulations currently employed for muscle tissue engineering, with a focus on their relevance and adaptability for modelling muscle spindles.

## 3D bioprinting strategies for muscle spindle modelling: Methods and bioinks

### 3D bioprinting methods

**Figure 1.**
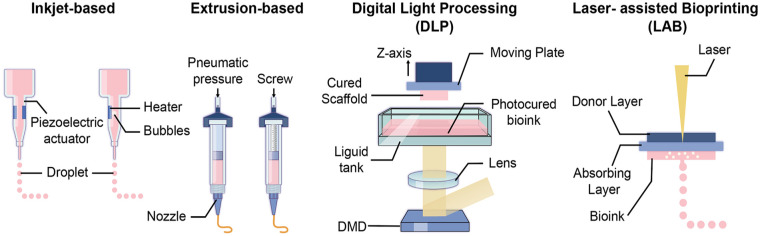
Illustration of four 3D bioprinting technologies. (a) Inkjet-based bioprinting: dispensing of tiny bioink droplets through the printhead using thermal or piezoelectric driving pulses. (b) Extrusion-based bioprinting: continuous deposition of cylindrical filaments using pneumatic or mechanical pressures. (c) Digital light processing bioprinting: stacking of cured layers using projected UV or visible light. (d) Laser-assisted bioprinting: use of focused UV or visible light beam to photopolymerize photosensitive bioinks. Illustration created by Medgy Design.

Currently, artificial skeletal muscles are mainly fabricated using four main types of 3D bioprinting technologies, including inkjet printing, extrusion printing, digital light projection (DLP) and Laser-assisted bioprinting (Figure 1).

The inkjet bioprinting system was initially developed from office inkjet printers, with the addition of a z-axis and the integration of biological components. The printing approach, commonly drop-on-demand (DOD) inkjet printing, involves dispensing tiny bioink droplets through the printhead using thermal or piezoelectric driving pulses.^83,84^ These pulses generate bubbles and transient sound waves within the nozzle, enabling precise, non-contact deposition of cell-laden bioinks onto a receiving substrate made of the desired material. In particular, thermal inkjet bioprinting is known for its compatibility with various cell types as it has minimal impact on cell viability post-deposition, while piezoelectric-based bioprinting may induce harmful frequencies that can cause cell membrane disruption.^85,86^

Fortunato et al.^87^ demonstrated the ability of piezoelectric-based inkjet bioprinting to generate aligned C2C12 myotubes using gelatin-based hydrogels. Similarly, Laternser et al.^88^ reported comparable results using human primary skeletal muscle cells, where they employed a photo-polymerisable gelatin methacryloyl (GelMA)-based hydrogel for effective myotube generation through inkjet bioprinting. Due to the nature of droplets driving mechanisms, small pores in the cell membrane can be induced transiently during the deposition process, which could potentially use to facilitate the delivery of genes and macroparticles, further supporting cell survival and growth. In general, inkjet bioprinting is valued for its high resolution (<50 µm), non-contact cell deposition and modifiable droplet size, making it suitable for patterning cell-laden constructs with fine structural features.^89^ Specifically, this technology is well-suited for in situ applications due to its ability to accurately deposit bioinks onto uneven or curved tissue surfaces, such as wounds.^90^ However, limited biomaterials, scalability and cost-effectiveness for large-scale tissue fabrication remain significant limitations. Although inkjet bioprinting enables high-speed microdroplets generation, it relies on discrete droplet deposition to create structures. As a result, the fabrication of larger constructs is time-consuming and often yields constructs with weak mechanical strength compared to other 3D bioprinting methods.^89^ In addition, a relatively low bioink viscosity and usually a cell density below 8 million cells/ mL are required for the smooth ejection of droplets, resulting in poor geometry and mechanical support of the bioprinted construct.^89,91^ To address this, post-deposition cross-linking is generally performed.^92^

Extrusion-based bioprinting, also known as filament-based or pressure-assisted bioprinting, allows the continuous deposition of cylindrical filaments into pre-defined CAD models using pneumatic or mechanical pressures (including screw- or piston-driven systems). This technique is the most explored bioprinting method due to its simplicity and broad bioink compatibility. One key advantage of extrusion bioprinting is that it allows for higher cell densities and more uniform cell distribution along the extrusion axis, which is especially important for muscle bioprinting, as it promotes better cell orientation and uniaxial alignment of myotubes.^66^ Since this technique does not rely on multiple intervening layers, the extruded constructs are generally self-supporting and require less post-processing. This allows for the production of micro-scale scaffolds in a relatively short time compared to inkjet and laser-assisted bioprinting, offering moderate to high fabrication efficiency – although not as high as that of digital light processing, which offers superior resolution.^93,94^

Extrusion-based bioprinting is also recognised for its scalability and capability for multi-material bioprinting. This can be achieved by integrating multiple nozzles within a single system, with customisable print heads and nozzle types tailored to produce specific outcomes.^58,95^ In recent years, kenics static mixers (KSMs) have been introduced in extrusion-based printing, which allows for the production of filaments with two or more channels of different biomaterials within each strand.^71,95^ This internal channelling approach mimics in vivo vascularisation by creating continuous branching channels, offering exciting possibilities for microvascular bioprinting and clinical applications.

However, it commonly requires bioinks with higher viscosity to ensure shape retention and minimise spreading of the bioink after extrusion. Consequently, the use of viscous biomaterials requires higher extrusion forces to overcome the increased resistance to flow through a small nozzle or needle, which can generate significant shear stress during the printing process and affect cell viability.^96^ Additionally, the resolution of standard extrusion bioprinting is relatively low (~100 μm), as it is determined by the nozzle’s diameter.^93–95^ It is challenging to maintain the viscosity of extrusion bioinks for printing fidelity while achieving optimal resolution.^97^ Bioinks that possess shear-thinning properties could address this limitation, by protecting cells from high shear forces during extrusion. Shear-thinning bioinks, also known as pseudo-plastic materials, have a lower viscosity during the printing process under high shear conditions but retain their original viscosity after deposition, thus preserving shape fidelity. For instance, decellularised ECM (dECM), sodium alginate and gelatin exhibit strong shear-thinning property, a characteristic often seen in higher molecular weight polymers. These hydrogels display non-Newtonian behaviour, allowing for high printing fidelity even at lower printing viscosities.^98,99^

Digital light projection (DLP) bioprinters were introduced in 2015,^100^ where a layer-by-layer fabrication technique is used by stacking cured layers using projected UV or visible light. The curing process occurs within a bath containing photocurable bioinks, where a digital micromirror device (DMD) projects a 2D pattern of light onto the bioink.^101^ One of the key advantages of DLP bioprinting is its fast fabrication time, as each entire layer is cured simultaneously. This process is repeated for subsequent layers, with a high-precision z-axis platform ensuring the application of a fresh bioink coating for each new layer. Unlike extrusion bioprinting, DLP bioprinting offers superior planar resolution (25–50 μm).^94^ This is because the size of the projected pixels from the DMD is generally smaller than most nozzle diameters used in extrusion bioprinting. As a result, DLP bioprinting enables the fabrication of more intricate and detailed geometries.^101^ While other bioprinting methods have been used to create microfluidic networks, DLP bioprinters are able to produce stronger tubular structures. This is because each cured layer has a larger contact area, meaning that the scaffold can withstand higher fluid pressures. By stacking slices of biomaterial on top of each other, it is also capable of creating negative space with 100% infill, providing greater control over the porosity of the constructs. This printing technique eliminates the need for sacrificial biomaterials to create hollow spaces, and enables the production of random internal structures, which could be beneficial for bioprinting tissues with defined 3D geometries, such as bones.^101,102^

Laser-assisted bioprinting (LAB) was first introduced to tissue engineering in 2004.^103^ Similar to DLP bioprinting, LAB is a non-contact, light-based manufacturing technique that offers a significantly higher resolution, typically around ~10 μm.^95^ However, this technique typically uses focused UV or visible light beam to photopolymerize photosensitive bioinks, such as modified alginate, gelatin, and fibrin.^55^ A key advantage of LAB is the ability to deposit bioinks at micro-scale resolutions without the need for nozzles.^104,105^ This feature effectively mitigates clogging problems often encountered when printing with higher cell density, enabling the isolation of cell aggregations within the bioink. Such high cell densities are crucial for muscle cell to align, fuse and differentiate, leading to the formation of better organised sarcomeres and enhanced functional contractility.^106^ Among bioprinting methods, LAB demonstrates exceptional cell viability during the printing process, as well as excellent cytocompatibility for multiple cell types. It is also capable of rapidly producing a smooth hydrogel surface in a layer-by-layer manner.^55,105,107,108^ Despite these advantages, LAB has limitations, including its high cost and limitations in transferring viscous bioinks to the substrate. Additionally, the process is relatively slow and inefficient for large-scale constructs, as only small amounts of bioink can be deposited per laser pulse.^55,105^

### Bioinks for skeletal muscle tissue bioprinting

Bioinks are highly hydrated and porous 3D carriers that consist of hydrogel solutions and ECM-based factors.^25^ In the literature, naturally-derived hydrogels are most commonly used in skeletal muscle bioprinting due to their high cell compatibility, controllable biodegradability, and low immunogenicity.^109,110^ Natural biomaterials are typically classified into two categories: ECM/protein-based and polysaccharide-based. Among these, protein-derived hydrogels offer the highest bioactivity as they are rich in various bioactive motifs.^109^ Increasingly, natural polymers are being integrated into hybrid bioprinting systems, where they are combined with synthetic materials like polyethylene glycol (PEG) to enhance mechanical strength and reduce batch-to-batch variations.^97,111^ For example, the degradability and elastic modulus of the hydrogel can be fine-tuned by modifying the chemical components of synthetic polymers.^109^ Photo-crosslinking is the most widely used chemical modification for structural stability, controlled degradation and tuneable mechanical properties. While frequently applied to synthetic hydrogels, many natural polymers such as gelatin methacryloyl (GelMA) can also be photocured. Additionally, sacrificial biomaterials (or fugitive bioinks) have been developed, primarily for the fabrication of vascular network. These biomaterials allow for the formation of perfusable microchannels within bioprinted muscle constructs, facilitating local oxygenation, nutrient transport and improving cell viability over extended culture period (Table 4).^54,71,77,95,112^

**Table 4.** Summary of natural bioinks used for skeletal muscle bioprinting.

Biomaterial	Key characteristic	Advantages	Disadvantages
Collagen	- Native ECM mimicry	- High biocompatibility - High cell adhesive properties - Good swelling properties	- Low mechanical strength - Expensive
Gelatin	- Thermo-reversible gelatin	- High biocompatibility - High cell adhesive properties - Cross-linkable - Biodegradable - Cost effective	- Low mechanical strength
Fibrin/ Fibrinogen	- Natural pro-angiogenic activity	- High biocompatibility - Good swelling properties - Cross-linkable	- Low mechanical strength - Expensive
Alginate	- Ionic crosslinking	- Cross-linkable - Cost effective	- Moderate biocompatibility - Lack of cell-adhesive properties - Low mechanical strength - Low swelling properties
Chitosan	- Anti-inflammatory properties	- High biocompatibility - Biodegradable	- Low mechanical strength - Slow gelation - Crosslinking can be cytotoxic
HA	- Myogenic signalling modulation	- High biocompatibility - Good swelling properties - Biodegradable - Cross-linkable	- Low mechanical strength
Matrigel	- Rich ECM proteins and growth factors	- High biocompatibility - High cell adhesive properties - Biodegradable	- Low mechanical strength - Expensive - Batch variability

### Protein-based hydrogels

For skeletal muscle tissue engineering, biomaterials derived from natural sources have been utilised in approximately 70% of all cell-laden bioprinting studies.^24,58,62,64,67,69^ Natural protein polymers such as collagen, gelatin, fibrin/ fibrinogen and silk are excellent candidate for cell-laden bioprinting due to their superior biocompatibility and biodegradability both in vitro and in vivo, their mechanical properties can also be modified via chemical crosslinking towards in vivo-like mechanical strengths. More importantly, natural polymers possess great biological complexity and intrinsic biochemical cues (e.g. cell binding sites) that are required for the recapitulation of tissue-matching ECM, contributing to efficient cell signalling, attachment, proliferation and differentiation, thereby resulting in high cell viability and functional maturation.^34,113^

#### Collagen

Collagen, the most abundant component of the skeletal muscle ECM, has been extensively utilised in skeletal muscle tissue engineering due to its excellent biocompatibility and ease of extraction. It constitutes 1%–10% of the dry weight of muscle tissue and contributes to the structural integrity of the muscle.^114^ Type I and III collagen are the predominant protein in the muscle epimysium and perimysium, with smaller amount of type V collagen present in the peri- and endomysium.^115,116^ With its abundant integrin-binding motifs, collagen it also known for promoting cell adhesion and proliferation. Collagen-based matrices, particularly those derived from collagen I, have been extensively used to support muscle growth, both in vitro and in vivo.^35,117–119^ Moreover, evidence shows that the myogenic potential of muscle satellite cells is better preserved in type VI collagen-coated surfaces, resulting in improved regeneration efficiency.^120^ In fact, type IV collagen is the core protein in the basement membrane of skeletal muscles and is found at substantial levels in the extracellular spaces of muscle spindles. This highlights the close anatomical connection between muscle spindles and the surrounding basal lamina, suggesting the potential role of type IV collagen in directing intrafusal fibre differentiation.^39,121^

Collagen-based bioinks are also known for their excellent swelling properties and minimal immunostimulatory effects, making them suitable for a range of tissue engineering applications. A high swelling ratio indicates increased water-holding capacity of a hydrogel, allowing scaffolds to retain more fluid and better resist shrinkage during storage or implantation – a favourable property for wound healing and muscle tissue engineering applications.^71,122^ However, the swelling property is closely linked to the mechanical and rheological properties of the hydrogel, as well as its degradation rate. Excessive swelling can result in poor structural fidelity and reduced mechanical strength.^122^ Collagen hydrogels typically exhibit relatively low tensile strength (~0.76 MPa)^24^ and are susceptible to rapid biodegradation through enzymatic activity. To address these limitations, collagen bioinks can be chemically modified, enhancing their stability and mechanical strength for more robust use in tissue engineering.^24,123,124^ For instance, the incorporation of alginate into 3D collagen blocks significantly enhanced cell proliferation and differentiation, outperforming collagen-only bioinks. This composite bioink also supported the differentiation of human adipose-derived stem cells (hASCs) into multiple lineages, including osteogenic and hepatic lineages.^125^

#### Gelatin

Derived from collagen, gelatin is frequently used in tissue engineering due to its inherent biocompatibility, ease of handling, low antigenicity and biodegradability. It can be derived from porcine (type A), fish (type A) and bovine (Type B) through acidic or alkaline hydrolysis of collagen. The differences in the extraction process lead to variations in the properties of the gelatin product, including its gelling strength (bloom number), viscoelastic properties, compressive strength, swelling and degradation properties.^126,127^ Porcine gelatin type A is also one of the most widely used natural polymers (~6% in cell-laden studies) for musculoskeletal applications,^24,67^ and in wound dressings due to its pro-angiogenic characteristics.^128,129^ As the denatured form of collagen, gelatin also retains its favourable cell attachment property and shares many of the structural and mechanical properties of the native ECM. For example, retaining the Arg-Gly-Asp (RGD) sequence enhances cell attachment, proliferation, and differentiation by promoting integral signalling, critical for tissue requiring strong cell-matrix interactions. Gelatin can be easily dissolved in aqueous solutions at physiological temperature and undergo gelation upon cooling due to its thermosensitive nature, which allows for controlled and thermos-reversible sol-gel transition, enabling the manipulation of gelatin-based scaffold into various shape and structures.^52^ Hu et al.^130^ developed a gelatin-based bioink exhibiting suitable shear-thinning properties and viscosity. By incorporating chitosan and α-cyclodextrin (α-CD) into the bioink, inclusion complexes form between α-CD and the hydrophobic side chains of chitosan, leading to pseudo-polyrotaxane (PPR) structures. The aggregation of these PPR side chains promotes physical crosslinking, resulting in the formation of supramolecular hydrogels. Owing to the non-covalent nature of these interactions, the hydrogel is dynamically reversible, exhibits good shear-thinning behaviour and is well-suited for bioprinting applications. This bioink also demonstrated good biocompatibility, tuneable mechanical strength and structural stability, maintaining integrity for up to 21 days under cell culture condition.

However, the thermal gelation of gelatin often leads to low mechanical strength and clogging when nozzles are involved (e.g. inkjet and extrusion bioprinting) due to its slow procedure. When modified into its more mechanically stable form, gelatin methacryloyl (GelMA), the stiffness of the bioink can be tailored to mimic the native microenvironment of the target tissue. This can be achieved by varying GelMA’s concentration, or the level of methacrylation and controlling the degree of UV photocrosslinking, which allows for fine-tuning of the bioink’s mechanical properties to meet the specific needs of different tissue types, from bone to cardiac tissue.^51,131,132^ To assess whether a 60-second exposure to 6.9 mW/cm² UV light (380–480 nm) affects cell viability, as suggested by Nichol et al.^133^ to be non-harmful, viability tests were conducted on gels photocrosslinked for 15, 30 and 60 s. Although hydrogels exposed to UV light for 60 s exhibited reduced viability compared to the other conditions on day 1, no significant differences were observed after 8 days. This may be attributed to enhanced proliferation and better access to nutrients in the bioprinted constructs compared to the bulk hydrogel controls.

A number of studies have shown that GelMA-based hydrogels at concentrations between 5% and 15% (w/v) effectively support cell adhesion, proliferation and differentiation of a wide range of cell types.^52,133–135^ Seyedmahmoud et al.^69^ utilised GelMA-alginate bioink to biofabricate 3D muscle construct using C2C12 cells, where GelMA was synthesised from gelatin type A and mixed with 0.5% (w/v) photoinitiator (2-hydroxy-4′-(2-hydroxyethoxy)-2-methylpropiophenone). It was demonstrated that the optimum concentrations for GelMA and alginate were 10% and 8% (w/v) respectively. However, direct comparisons should be interpreted with caution. Due to the absence of reported degree of methacrylation (DoM) and crosslinking conditions, the GelMA composition was assumed to fall within commonly used ranges (e.g. 5–15% w/v, medium to high DoM). These parameters significantly affect mechanical properties, stiffness of the hydrogel and cell behaviour. Similar findings was reported by Shao et al.^136^ and García-Lizarribar et al.,^68^ where GelMA-alginate blend supported effective cell spreading, viability, proliferation and differentiation.

Costantini et al.^137^ investigated the effect of GelMA hydrogel stiffness on C2C12 differentiation by tuning GelMA concentrations, where 3%–4% GelMA hydrogels provided favourable stiffness for myoblasts and promoted significant myotube formation in 2 weeks. Typically, the strength of GelMA requires further optimisation as it displays relatively low viscosity at physiological temperatures. This limitation is particularly important in extrusion-based bioprinting, where viscosity directly affects printability, structural fidelity and shear-thinning behaviour. Thus, studies have incorporated other biomaterials such as alginate and Hyaluronic acid (HA) into GelMA-based bioink to improve the structural fidelity and mechanical performance such as shear-thinning properties of hydrogels, meeting the criteria for the bioprinting process while obtaining its bioactivity.^68,69^ Similarly, Boularaoui et al.^72^ reported that 2% GelMA supported optimal cell viability and differentiation using C2C12 cells, however, the hydrogel exhibited poor print fidelity. To improve the printability and rheological properties of the GelMA hydrogel, they developed GelMA-gold nanoparticles and GelMA-MXene nanosheets hydrogels, which demonstrated enhanced printability and shear thinning behaviour. Notably, the addition of gold nanoparticles significantly increased the fusion index of C2C12 myoblasts. Additionally, GelMA can be combined with other biomaterials, such as PF-127 and agarose, to form microchannel networks within thicker bioprinted constructs.^71,74,138^ Once the sacrificial layers are removed, the resulting hollow vascular network allows for perfusion and facilitates efficient mass transport throughout the construct.

#### Fibrinogen

Fibrinogen is a soluble plasma glycoprotein primarily produced by the liver and can be found in the bloodstream. During blood clotting, it is cleaved by thrombin to form insoluble thrombin, which assembles into a fibrous network.^25,139^ As key components of the natural coagulation process, fibrin and fibrinogen play a critical role in wound healing by preventing excessive bleeding and modulating inflammatory responses.^25,52,140^ Fibrinogen-based hydrogels exhibit excellent biocompatibility, making them an ideal matrix for cell adhesion, proliferation and regeneration. This is due to the presence of cell-binding motifs that facilitate interactions with integrins on the cell surface.^25,141–143^ Additionally, fibrinogen scaffolds are highly dynamic, as they can be remodelled by cells through fibrinolysis – a proteolytic process that enables the complete degradation of the constructs. This property not only ensures superior biodegradability but also supports long-term cell adhesion, tissue replacement and functional integration of the grafts.^143,144^

It should be noted that fibrinogen on its own exhibits low viscosity, limited mechanical strength and a rapid degradation process prior to crosslinking, which significantly reduces its printability and structural fidelity in extrusion-based bioprinting systems.^25,143^ However, its low viscosity makes it suitable for inkjet-based bioprinting applications, as fibrinogen solution can be ejected as tiny droplets without clogging and crosslinked immediately following deposition. Plasmin inhibitors can be used to effectively delay the degradation of fibrin network from days to weeks, both in vivo and in vitro.^144,145^ However, this could also result in decreased cell proliferation, likely due to a reduction in the production of collagen and elastin, which are essential for maintaining tissue structure and function.^144^ Crosslinking modification is one of the most widely used strategies to enhance the mechanical properties of fibrinogen. By combining fibrinogen with other biomaterials that exhibit better gelation properties, the bioprinting process and shape fidelity post-deposition can be significantly improved.^143^ Suitable candidates for this include alginate, gelatin and hyaluronic acid (HA). Alginate and fibrinogen hydrogels have been utilised in 3D bioprinting, demonstrating high bioactivity, with alginate providing essential mechanical support.^146,147^ Xu and Wang^148^ developed a hybrid bioink composed of alginate, fibrinogen and gelatin to fabricate microfluidic channels, which effectively promoted the growth and proliferation of adipose-derived stem cells (ADSCs). The incorporation of alginate also plays a critical role in extending the degradation time of fibrin through ionic and enzymic crosslinking, further enhancing the stability and functionality of the scaffold.^143,148,149^

In skeletal muscle tissue engineering, fibrinogen-based hydrogels supplemented with hepatocyte growth factor (HGF) have been developed to promote the regeneration of C2C12 cells, in VML models.^32^ Similarly, Matthias et al.^150^ and Thorrez et al.^151^ observed comparable outcomes of fibrin hydrogels in their studies using muscle-derived stem cells (MDSCs) and primary human skeletal myoblasts, respectively. Additionally, Ronzoni et al.^61^ compared the myogenic and differentiation potential of 3D bioprinted C2C12 cells in commercially available bioinks including CELLINK^®^ GelMA A, CELLINK^®^ GelXA FIBRIN and CELLINK^®^ FIBRIN hydrogels. The results suggested that CELLINK^®^ FIBRIN consisting nanofibrillated cellulose, alginate and fibrinogen induced the highest level of myotube differentiation, alignment and gene expression.

#### Silk fibroin

Silk fibroin (SF) is a fibrous protein primarily obtained from *Bombyx mori* silkworm silk, increasingly popular in 3D bioprinting due to its outstanding mechanical strength, adjustable biodegradability, low immunogenicity and high biocompatibility.^51,152^ It features highly organised β-sheet structures that provide significant stability and elasticity, making it versatile for processing into various forms, including hydrogels, films and sponges.^153,154^ Its unique molecular structure mainly comprises fibroin as the core protein, surrounded sericin proteins that act as a binding layer, which is usually extracted to improve biocompatibility and reduce allergic responses in vivo. Silk fibroin can transition from random coils and alpha-helices to beta-sheet crystalline structures, a process crucial for forming stable hydrogels. It also enables controlled porosity at micro- to macro- scales, facilitating cell infiltration and nutrient exchange within the bioprinted constructs.

Silk-based hydrogels have been shown to support excellent cell viability and proliferation in various cell studies.^153,155–157^ The ability of silk-based systems to form porous scaffolds with hierarchical organisation and maintain excellent mechanical properties are particularly useful for musculoskeletal applications.^154,156,158,159^ For instance, SF-based hydrogels designed to mimic bone structures can enhance osteogenic differentiation. Porous 3D structures have been shown to support cell spreading, proliferation and differentiation compared to solid scaffolds. Chaturvedi et al.^155^ examined the impact of various silk fibroin (SF) types on primary human skeletal muscle myoblasts. Their findings indicated that porous SF-based scaffolds derived from *Bombyx mori* silkworms promoted myotube formation, alignment and maturation. In contrast, scaffolds derived from A*ntheraea mylitta* and *Antheraea assama* resulted in shorter and less oriented myotubes. The study suggested that scaffold architecture and elasticity played a more critical role in myotube formation than fibroin composition.

By blending silk fibroin with other biomaterials, it can also be tailored for muscle 3D bioprinting. Kamaraj et al.^157^ developed SF-based bioinks reinforced with silk microfibre for VML study. Gelatin and short silk microfibres were incorporated into the SF hydrogel for extrusion-based 3D bioprinting, resulting in improved mechanical properties ad cell-matrix interactions, consistent with previous studies.^160,161^ More importantly, microfibre-reinforced SF hydrogels promoted greater C2C12 myoblast adhesion and myotube alignment, highlighting the positive role of SF microfibres in muscle growth and regeneration. Moreover, the degradation rate of silk fibroin is relatively slow, generally taking up to a year or more and degrades via proteolysis. This also makes it ideal for applications requiring long-term structural support, such as muscle and bone tissue engineering and regeneration.^152^

### Polysaccharide-based hydrogels

#### Alginate

Alginate, a natural biomaterial derived from brown algae (*Phaeophyceae*), is widely used in skeletal muscle bioprinting due to its biocompatibility, low cytotoxicity, affordability and ease of handling.^162,163^ Structurally similar to the glycosaminoglycans found in the native ECM, alginate consists of linear polysaccharides that are composed of two monosaccharide units, namely mannuronic acid (M) and guluronic acid (G). The ratio and arrangement of these units determine the strength and stability of alginate gel. Higher molecular weight and an increased length of G-blocks result in stiffer gels with greater viscosity and enhanced gelation properties compared to M-rich alginate gels. One of the key features of alginate is its capacity to form hydrogels via ionic crosslinking with divalent cations, such as Ca²⁺ and Mg²⁺.^109^ This process enables fast gelation by crosslinking guluronate blocks in the polymer chains.^162,164^ Calcium chloride (CaCl₂) baths are commonly used for crosslinking alginate hydrogels. Although slower gelation rate is believed to form structures with greater technical integrity and uniformity, having a fast gelation in 3D bioprinting contribute to better printability and reduced processing time.^52,165^ However, it has been noticed that alginate gels cross-linked with CaCl₂ tend to exhibit reduced long-term stability, especially under physiological conditions.^166^ This occurs as the divalent ions gradually release into the surrounding media, causing the hydrogel to dissolve and lose mechanical strength. In some cases, pre-crosslinking of the alginate gel with CaCl₂ can be performed, but the CaCl₂ concentration should be minimised to reduce cytotoxicity.

While alginate has been widely used in bioprinting with various cell types and has shown a protective effect against pressure applied during printing, its bioactivity is relatively low. Consequently, alginate is often combined with gelatin or fibrin to enhance cell adhesion and functionality, promoting cell-matrix interactions.^51,167,168^ By tuning the viscosity of alginate, biomimetic mechanical strength can be achieved when integrated with other biomaterials. For instance, Bolívar-Monsalve et al.^71^ utilised a bioink blend composed of 3% GelMA and 3.5% low-viscosity alginate blend to promote muscle cell spreading. According to Seyedmahmoud et al.,^69^ although 8% (w/v) alginate was found to induce more profound myotube proliferation, metabolic activity and differentiation in GelMA-alginate hydrogels, myotubes cultured in 6% (w/v) alginate exhibited better cell spreading at day 7.

Yu and Zhang et al.^169,170^ conducted similar experiments, examining alginate concentrations ranging from 2% to 6% (w/v). They found that higher alginate concentrations decreased cell viability, degradation rate, pore size and permeability, likely due to increased viscosity and resulting shear stress during the printing process. Based on their investigation of ECM matrix deposition, they concluded that a 4% (w/v) alginate concentration provides optimal cell viability and matrix deposition. Thus it is essential to consider the nature of the biomaterial when adding complexity to the bioink composition, as the mechanical and rheological properties can vary significantly depending on whether alginate is used alone or blended with other biomaterials. These variations can affect cell spreading, survival, proliferation and metabolic activities. Careful optimisation of bioink concentration, scaffold design and printing parameters is necessary for each formulation to ensure optimal outcomes.

Additionally, alginate-based scaffolds can be loaded with GFs such as VEGF, hepatocyte growth factor (HGF), insulin-like growth factor-1 (IGF-1) and stromal cell-derived factor-1 (SDF-1) to support local delivery. The addition of SDF-1-encapsulating alginate microspheres into collagen-based hydrogels have shown to enhance the delivery of SDF-1 and recruitment of angiogenic cells, promoting angiogenesis and muscle regeneration in an ischaemic skeletal muscle model.^171^

#### Chitosan

Chitosan-based hydrogels are highly biocompatible and biodegradable due to its unique bioactivity and structural properties. Derived from deacetylated chitin – a natural linear polysaccharide commonly found in the exoskeletons of invertebrates such as insects and crustaceans^172^ – chitosan exhibits versatile biological functions. The functional amino groups in chitosan give it a positive charge under acidic conditions, allowing for haemostatic and mucoadhesive activities and facilitating interactions with cell membrane by recognising negatively charged components.^65,173^ These interactions can alter the permeability of cell membrane and inhibit microbial intrusion, providing chitosan with notable antimicrobial properties.^172,174^ Chitosan’s amino groups also contribute to its ability to form complexes and its solubility in aqueous environments, though this solubility is limited to acidic solutions (pH < 6.5).^25,172^ To enhance cytocompatibility, chemical modifications can be made, such as by carboxymethylation, which introduces carboxymethyl groups (-CH2-COOH) into the structure, improving chitosan’s solubility across a wider pH range (both acidic and neutral pH).^25^

Chitosan can be prepared as hydrogels, sponges and films, each offering different features suited for various applications. For example, chitosan sponges are frequently used for wound dressing due to their ability to function as absorption pads. They have also found applications in tissue engineering, particularly as fillers for bone constructs.^172,175^

Chitosan-based films are typically nanostructured and porous, also shown potential for wound healing. These films have been shown to accelerate wound healing and are suitable for use in drug delivery systems.^172,176,177^ Leung et al.^178^ also found that chitosan-based films and nanofibers effectively promoted the myogenic differentiation of muscle progenitor cells, leading to increased expression of Myf5, Myf6, myogenin and MHC.

For muscle tissue engineering, chitosan-based hydrogels are mostly studied. Hajiabbas et al.^179^ developed chitosan-gelatin hydrogels to create scaffolds with mechanical properties similar to muscle tissues, where significant increase in cell viability and proliferation of muscle-derived cells has been reported in hydrogel of chitosan (2%) and gelatin (6%) concentrations. However, chitosan-based scaffolds typically have poor shape fidelity,^65^ which can be improved by introducing side chain such as polyethylene glycol (PEG) to enable physical crosslinking.^130^ It can also be prepared with other bioinks, for example, chitosan-alginate blends have shown the ability to promote the proliferation of human adipose derived stem cells (hASCs) while preserving high shape fidelity.^180^ By fine-tuning their rheological and mechanical characteristics through crosslinking, these bioinks are highly effective for controlled drug delivery, ensuring sustained release and improved therapeutic outcomes.^181^

## Recent advances in the molecular characterisation of muscle spindles

Understanding the anatomy, formation and function of muscle spindles are the first steps of establishing functional and biomimetic in vitro 3D models. Recent studies have provided invaluable insights into the unique cellular and extracellular environments of intrafusal fibres, sensory neurons and capsule cells. These molecular discoveries offer a promising direction for enhancing the design of bioinks for skeletal muscle tissue bioprinting, particularly in replicating the complex architecture and functional properties of muscle spindles. For instance, the identification of specific biomarkers associated with intrafusal muscle fibres – such as the distinct ECM components and cellular signalling pathways – can inform the selection of materials for bioinks that more accurately mimic the microenvironment of muscle spindles. This, in turn, would enhance more precise cell-cell and cell-matrix interactions, which are crucial for developing in vivo-like bioprinted muscle spindles.

Unlike extrafusal fibres, the survival and differentiation of the muscle spindle depends on the establishment of the neuromuscular connection between two types of proprioceptive sensory afferents and primary myotubes, which gives rise to the intrafusal-specific myosin heavy chain (MyHC) profile.^11,45,182–184^ The deep anatomical location of muscle spindles also poses challenges for their accessibility and investigation. Due to their small structure and proximity to surrounding extrafusal fibres and connective tissues, isolating muscle spindles requires advanced dissection and microscopic skills. Although specialised histological, immunohistochemistry and electrophysiological techniques can be used to identify muscle spindles, their detection remains difficult because of their low density per muscle mass, especially for force-generating muscles that are responsible for rapid locomotion, such as gastrocnemius. Consequently, studies characterising muscle spindles are more advanced in other mammals such as rats, pigs, chick, and sheep, and that the molecular events underlying spindle formation and function remain less understood. The pathophysiology of muscle spindles, compared to extrafusal fibres, has also been overlooked in therapeutic development, as often they do not represent the primary symptoms.

**Figure 2.**
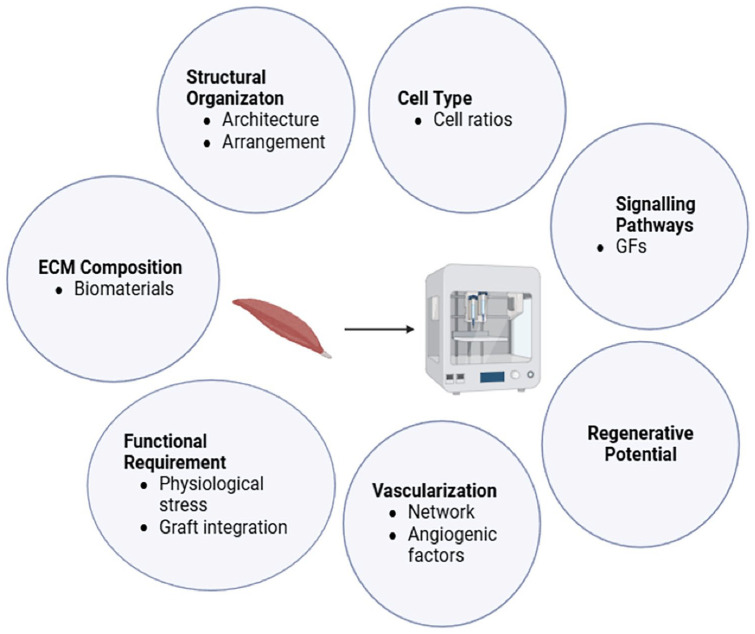
Biological insights guiding 3D bioprinting design and optimisation. The diagram highlights the role of biological insights in determining: (a) Structural Organisation: the spatial arrangement of cells within the bioprinted construct to mimic native tissue architecture; (b) Cell Type: selection of appropriate cell types and ratios to ensure the desired functionality; (c) Signalling Pathways: incorporation of biochemical cues to regulate cell behaviour, differentiation and tissue development; (d) Regenerative Potential: how the construct’s design supports tissue regeneration, including factors that influence cellular proliferation and repair; (e) Vascularisation: strategies for promoting blood vessel formation within the construct to ensure nutrient and oxygen supply; (f) Functional Requirement: how the design meets the mechanical and biological needs of the target tissue, such as load-bearing capacity or electrical conductivity; and (g) ECM Composition: the integration of ECM components to support cellular attachment, migration, and differentiation.

The section below summaries the muscle spindle’s specific fibre morphology, structural arrangement and myonuclear populations, the mechanisms regulating the specification of functionally distinct intrafusal fibres, including novel molecular markers and discoveries. These key biological and mechanical features specific to muscle spindle are necessitated for guiding model development and the validation tissue engineered scaffolds, ensuring the model mimics tissue’s native architecture and function effectively (Figure 2). By coupling with advanced biofabrication technologies, 3D bioprinted muscle constructs have more defined matrix organisation and molecular composition, which overcomes the simplified microenvironment of traditionally engineered intrafusal fibres.

### Anatomy

**Figure 3.**
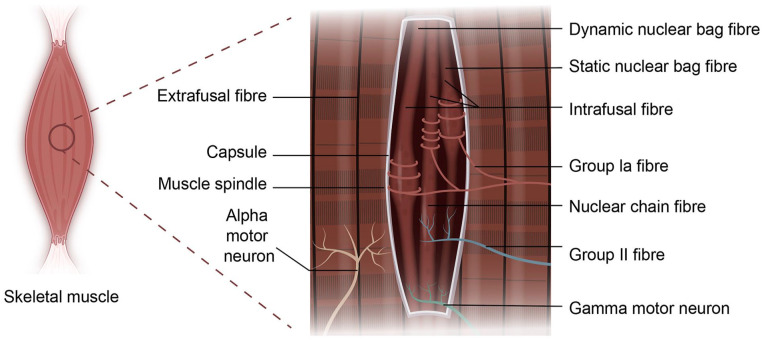
The anatomical structure of muscle spindle. Illustration created by Medgy Design.

Muscle spindles are complex sensory organs embedded in skeletal muscles and run parallel to extrafusal muscle fibres. They comprise of small bundles of specialised intrafusal fibres, which receive their specific sensory and motor innervations in a ‘fusiform’ capsule.^183,185–188^ The multi-layered capsule creates a fluid-filled periaxial space with a unique extracellular matrix (ECM) composition as secreted by the capsule cells, which also serve as a diffusion barrier and provide mechanical support.^189^ This surrounding connective tissue also forms a protective sheath, contributing to the maintenance of spindle sensitivity and ensuring that the proprioceptive feedback remains accurate (Figure 3).

In conditions such as muscular dystrophy, fibrosis or other neuromuscular disorders, the spindle capsule is often affected, with a notable thickening/ stiffness of the capsule due to excess deposition of ECM which leads to impaired proprioception.^6,190,191^ Additionally, capsule cells contribute to the production of ECM proteins, including versican (VCAN), elastin, collagen IV and matrix metalloproteinases (MMPs), as well as signalling molecules in the extracellular space,^17,192,193^ where collagen IV has been recognised as a marker for spindle ECM. Recent studies are beginning to provide deeper insights into the molecular characteristics and functions of the spindle capsule, suggesting that the expression of VCAN in the extracellular space within the capsule region could be a potential marker specifically for capsule ECM.^17^ In particular, the protein GLUT1, has been newly identified as being upregulated at both protein and RNA levels in the outer capsule, which connects to the peripheral nerves as a part of the perineurial sheath.^194^

In contrary to the surrounding extrafusal fibres, the morphology of intrafusal fibres appear much thinner and shorter as they have very little role in force generation.^185,195,196^ Typically, muscle spindles in humans contain 8–20 intrafusal fibres, while a single mouse muscle spindle may contain up to 5 intrafusal fibres.^10,197^ Approximately 0.4 and 1.7 muscle spindles were reported per gram of human gastrocnemius and flexor hallucis longus muscles, respectively.^198^ However, its mean density is generally higher in muscle types that are capable of fine motor adjustment or postural control.^186,198–201^ The differences in the composition between intrafusal and extrafusal fibres have only been investigated in recent years. Bornstein et al.^17^ carried out thorough proteomic analysis and revealed 24 proteins that were exclusively expressed in intrafusal fibres, but not in extrafusal fibres. These proteins were mainly myosins and ECM proteins such as elastin and versican. Up to 40 proteins were differentially expressed in both the muscle spindle and the surrounding neuronal tissue, and had almost no expression in the extrafusal fibre samples, suggesting a distinct protein expression pattern in the muscle spindle and its associated neuronal components.

Within the inner capsule, intrafusal fibres exist in three distinct subtypes with different functions: the larger ‘dynamic’ nuclear bag fibres, the smaller ‘static’ nuclear bag fibres and the small nuclear chain fibres.^10,185,187,202^ The dynamic bag fibre, typically known as the nuclear bag 1 fibre, is involved in signalling the velocity of muscle stretch, tension and length, contributing to the dynamic stretch reflex during rapid locomotion. It has the lowest contractile properties due to the absence of an M-line in the sarcomere and the presence of smaller and fewer mitochondria. In contrast, the static bag fibre, or nuclear bag 2 fibre, signals the absolute length of the muscle, aiding in posture coordination and muscle tone. It exhibits intermediate contraction rates and oxidative enzyme activity and possesses a central M-line. These nuclear bag fibres are characterised by their densely clustered nuclei in the equatorial region, forming a central bulge that extends towards the polar ends and beyond encapsulation, where they attach to the connective tissue of the spindle capsule.^203^

Unlike the nuclear bag fibres, the nuclear chain fibres are encapsulated in the spindle capsule entirely. They are smaller in length and contain fewer nuclei in a row within a single chain, as they typically consist of more and larger mitochondria to support their greater contractility and glycolytic oxidative enzyme activity.^10,204^ Additionally, nuclear bag 1, nuclear bag 2, and nuclear chain fibres display varying intensities of myofibrillar histochemical ATPase reactivity. These differences in reactivity can be utilised to distinguish the three intrafusal fibre subtypes. Specifically, nuclear bag fibres typically exhibit acid-stable ATPase, while nuclear chain fibres are detectable only under alkaline conditions.^202,205^ Moreover, differences in myosin composition among intrafusal fibre subtypes have also been reported. Myosin light chain 2 (MYL2), was found to have an exclusive expression in nuclear bag fibres and was absent in nuclear chain fibres, extrafusal fibres and muscle-neuron interfaces.^17^

### Innervation

Understanding the molecular markers within muscle spindles and their associated proprioceptive neurons is essential for characterising muscle spindles in vitro. The sensory and motor innervations of muscle spindle have been well-described since the 1950s,^203,204,206–208^ however, the mechanisms underlying this process of mechanotransduction remain less understood. Two types of sensory afferents, type Ia and type II, innervate the central region of the intrafusal fibre. These are also termed ‘primary’ and ‘secondary’ proprioceptive sensory neurons based on their conduction velocity.^187,193,209^ Type Ia afferents coil around the equatorial portion and form annulospiral wrappings (ASWs), which register the change in muscle length and velocity as primary sensory feedback for proprioception. In contrast, type II afferents are more sensitive to the static length, they typically form flower spray endings closer to the polar ends if present. Specifically, every muscle spindle is innervated by a single type Ia afferent, with one spiral per intrafusal fibre. The endings of type Ia afferents are larger in diameter and exhibit greater velocity in axonal conduction in comparison to type II afferents, hence considered as the dynamic axon.

The type II afferents terminate on intrafusal nuclear bag 2 and chain fibres at polar ends, forming flower-spray endings (FSEs) with smaller spirals. These axons primarily signal static information.^203,204,206,209^ The higher firing frequency of sensory afferents indicates a greater degree of stretch or a change in muscle length, corresponding to static and dynamic responses, respectively. Inter-species differences are also observed in the sensory innervation of intrafusal fibres. While it is uncommon to detect type II afferents in mice, both type Ia and II afferents in humans can signal dynamic and static status during stretching, with no ASW endings found at the primary terminals.^12,208,210^

The importance of proprioceptive sensory neurons in muscle spindle development was recognised in the late 20th century.^211–213^ Early genetic studies and knockout models discovered that sensory neurons not only innervate muscle spindles, but also play a crucial role in the formation and maturation of spindle fibres. For example, neurotrophin-3 (NT-3), a critical neurotrophic factor for the survival and maintenance of proprioceptive neurons, serves as a chemoattractant, guiding the proper outgrowth and terminal branching of sensory axons.^213–215^ Mice lacking NT-3, its receptor, tyrosine kinases receptor (TrKC) or Runnx3 exhibit severe ataxia and fail to develop muscle spindles as the axons were not able to innervate the target muscles. Impaired stretch reflex arc circuit was also reported due to disconnection between sensory and motor neurons. Additionally, the absence of NT-3 results in abnormal axon termination with inappropriate loci.^213,214,216,217^

Piezo2 is another established marker for mammalian proprioception, particularly in sensory neurons involved in mechanotransduction.^14,218–220^ It is a mechanically activated cation channel that is predominantly expressed in the sensory endings of proprioceptive axons, which innervates mechanoreceptors such as muscle spindles and Golgi tendon organs. Studies have shown that the Piezo protein is essential for the regulation of proprioceptive feedback and the stretch-sensitive afferent activity of muscle spindles, allowing the detection and transmission of sensory information in response to mechanical stimuli.^14^ Ablation of Piezo2 expression in mice leads to significant gait disturbance, impaired coordination and mechanically insensitive neurons.^219,221^ Moreover, Piezo2 was believed to be the principle mechanosensor for muscle spindles. It is responsible for the initiation of afferent depolarisation by facilitating the release of glutamate-containing synaptic-like vesicles via vesicular glutamate transporter 1 (VGLUT1). The release of glutamate enhances the sensitivity of spindle afferent.^220^

Bornstein et al.^17^ examined the expression of tdTomato in a Piezo2 knockout mouse and reported its expression in sensory neurons, intrafusal fibres and capsule cells, except from the proprioceptive sensory endings. This finding aligns with earlier studies that identified Piezo2 expression in nuclear bag fibres, the neuromuscular junction and the lateral contractile regions of the spindle fibre, while it was absent in the central region. This suggests that the presence of a specialised fibre compartment within the neuron-muscle contact region. With advancements in 3D modelling of intrafusal fibres, increasing the expression of Piezo2 and NT-3 could enhance the specificity of the culture microenvironment and help researchers better understand neuron-muscle interactions, such as the mechanosensory impairment via muscle spindles in disease.

Muscle spindles also receive efferent motor control from the CNS via fusimotor or motoneurons. The most prevalent type is known as the gamma motor neurons (γMN), and they make up approximately 30% of all fusimotor innervation originating from the spinal cord. These are structurally and functionally different from the alpha motoneurons that innervate extrafusal fibres.^187,222^ These axons can be distinguished using ramp-and-hold experiments to ascertain their static or dynamic function.^223^ γMN possesses smaller axons with extensive branching that selectively innervates the striated regions at the poles of the intrafusal fibres, which mediates the discharge frequency of sensory afferents by contracting at the polar ends, thereby causing the noncontractile central segment to stretch so that the muscle tension can be restored.^208,224^ During dynamic muscle activity, axons of dynamic γMN predominantly innervate the intrafusal nuclear bag 1 fibre, eliciting excitatory spikes at the onset and conclusion of the stretch phase. Alternatively, static γMNs are specialised to monitor and adjust to the constant muscle length.^197,225^ Previous studies have demonstrated the dependence of γMN on muscle spindle-derived GDNF for postnatal survival and function in the peripheral nervous system.^226–228^ GDNF plays a key role in the development and maintenance of neuron-muscle interactions and is dysregulated in ageing and various diseases.^229–231^ Later studies utilised its receptor, Gfrα1, to trace γMNs from birth and explore spindle-specific fusimotor activity during postnatal development.

### Formation

In humans, the formation of muscle spindles is initiated as early as 11 weeks during the foetal stage and continues to develop after birth.^232–236^ The fine structure of the developing spindle tissue can be observed after the ninth week of gestation, where a smaller group of myoblasts is surrounded by layers of flattened mesenchymal cells, with an intimate association with nerve fibres.^235^ The spindle fibre becomes well-differentiated by the week of 22.^233,235^

According to Zelena,^182,237^ the initial development of the muscle spindle depends on the early contact between the Ia afferent terminals and myocytes, which enables the subsequent differentiation and the formation of capsule and periaxial space. The first detection of spindle formation in foetal rats was by day 19.5, marked by the aggregation of nuclei in the contact region of the neuromuscular connection, indicating the formation of the first intrafusal nuclear bag fibre.^10,238^ Following the extension of intramuscular nerves, the perineural epithelium elongates and surrounds the innervated myotube, forming the early shape of the spindle capsule.

By day 20, the fusion of the second myotube takes place and is considered to have originated from satellite myoblasts.^239^ The myotubes remained functionally immature at birth, where the encapsulated myoblasts beginning to extend towards the poles and forming the first intrafusal nuclear chain fibre by postnatal day 1. This is followed by a series of morphological and physiological changes of the innervating sensory afferents while the muscle mass increases, thereby acquiring the ability to respond to stretch. In more detail, the development and maintenance of the spindle depend heavily on the dual innervation of sensory and motor neurons. Intrafusal fibres retain features characteristic of immaturity, such as the expression of PAX3, Myf5 and neonatal MyHCs. There are also greater numbers of satellite cells within the muscle spindle compared to extrafusal fibres,^240^ which may indicate a distinct regenerative potential when compared to their neighbouring extrafusal muscle.^21^

Neuregulin-1 (NRG-1) signalling is one of the most important signalling pathways in the development and maturation of intrafusal muscle fibres. NRG-1, predominantly secreted by sensory neurons, binds to ErbB receptors on muscle progenitor cells and triggers the downstream expression of Egr3, a transcription factor critical for intrafusal fibre specification. It has been demonstrated that muscle formation is heavily influenced by the signalling between sensory and motor neuron terminals and muscle cells. Notably, an increase in ErbB receptors has been observed during the formation of neuromuscular junctions (NMJs).^241,242^ In particular, ErbB2 is required for the developmental process of intrafusal fibres. Andrechek et al.^243^ demonstrated a positive correlation between ErbB2 levels and the number of muscle spindles, with ErbB2 gene ablation leading to significant proprioceptive deficiencies in mice. Additionally, Leu et al.^242^ explored the role of ErbB2 in NMJ development and reported normal afferent-myotube interactions during the early stages of muscle spindle development. However, the maturation of spindle fibres was inhibited in the absence of ErbB2. Furthermore, ErbB2-deficient mice exhibited reduced synaptic transmission efficiency, as indicated by a significant decrease in acetylcholine receptor (AChR) density at the NMJs.

NRG1-deficient mice exhibited severely impaired intrafusal fibre morphology and differentiation, as reported by Hippenmeyer et al.^45^ This was characterised by the absence of downstream Egr3 expression and annulospiral branching at proprioceptive afferent terminals. These findings align with earlier research by Tourtellotte and Milbrandt,^244^ which showed that Egr3-deficient mice lacked muscle spindle and developed gait ataxia, perinatal death and resting tremors. Together, these studies highlight the importance of neural inputs (i.e. Ia afferent innervation) in initiating myogenic differentiation and intrafusal fibre development.

Immunocytochemical evidence has also demonstrated the co-expression of alpha cardiac-like myosin heavy chain (MHC) and phosphorylated ErbB2 receptors in in vitro-generated intrafusal fibres, using monoclonal BA-G5 and phospho-ErbB2 antibodies.^36^ Moreover, it has been proposed that the co-expression of Egr3 with BA-G5 MHC, following NRG-1 signalling, can serve as a marker for identifying nuclear bag fibre morphology.^11,45^ Therefore, quantifying NRG-induced expression patterns and the profiles of intrafusal-specific myosin heavy chain (MyHC) isoforms provides a reliable method for assessing the development of intrafusal fibres.^17^

## Design criteria for muscle bioprinting

**Figure 4.**
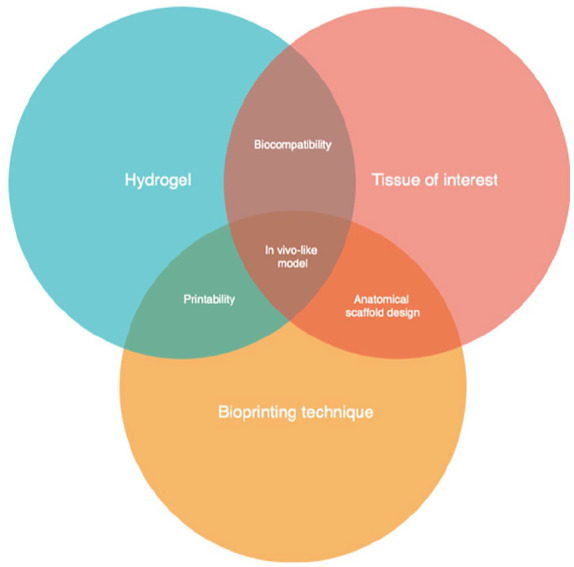
Three Key factors to consider for 3D bioprinting design. This figure illustrates the interconnection between three critical factors – hydrogel formulation, tissue of interest, and bioprinting technique. The overlaps between these factors emphasise the importance of carefully considering how each element contributes to the development of a biomimetic and functional bioprinted construct with tuneable mechanical properties. Achieving an optimal balance between these factors is essential for creating tissue-engineered models that closely mimic native tissue behaviour and function.

3D bioprinting technology introduces additional complexities, requiring careful consideration of factors such as the selection of suitable bioinks, cell sources, bioactive molecules and the optimisation of techniques and parameters involved in the printing process (Figure 4).

Cell- or bioactive molecule-encapsulating bioinks are the foundation of 3D bioprinting, it provides an ideal living microenvironment with defined geometries for the desired tissue type to spread, grow and differentiate. To determine the suitability of a bioink for cell-laden bioprinting, it is crucial to first evaluate its ability to maintain cell viability. Considering the biology of muscle spindles, a complete bioink formula for intrafusal fibre bioprinting should accurately represent the natural microenvironment of the muscle spindle, for example, the inclusion of ECM components such as collagen, glycosaminoglycans (GAGs), fibrin, elastin, basal lamina molecules (e.g. laminin and agrin) and growth factors would provide biomimetic intrinsic features, further supporting the specification of intrafusal fibres.^57,245,246^ The selection of bioprinting methods is equally important. While extrusion-based bioprinting provides the flexibility to utilise a broader range of bioinks and cell types, inkjet bioprinting, DLP and LAB are advantageous for ensuring high cell viability during the printing process. These techniques typically employ low-viscosity bioinks and minimise shear stress – especially DLP and LAB, as they operate without direct nozzle contact.^97,247^

However, the primary obstacle in muscle 3D bioprinting lies in developing a bioink that provides a biocompatible environment while maintaining good printability and mechanical properties. Printability generally refers to the ability of a bioink to preserve its shape and align with the theoretical dimensions of the design after deposition.^97^ Most biomaterials that support biological events (e.g. collagen) require additional modifications to improve their printability as they typically inherit weaker rheological properties and poor shear-thinning behaviour. To overcome these limitations, different gelation mechanisms (e.g. temperature, photo-crosslinking and chemical modifications) and biomaterials (stiffer or softer) can be incorporated into the system to improve the rheological properties of the bioink. There are different methods proposed in previous studies to quantify the geometric fidelity of bioprinted constructs, focusing on parameter such as the diameter, uniformity, porosity and stability of printed hydrogels.^92,97,248,249^ These methods can be selected based on the specific bioprinting context.

Increasing the viscosity of hydrogels also enhances shape retention and printing fidelity in bioprinted constructs due to a higher polymer concentration. However, this also affects cellular performance, as greater viscosity leads to greater resistance for hydrogels to flow during deposition and limits cell spreading within the construct. Although hydrogels have high flexibility and water content, which provides a cushioning effect and shield cells from the shear forces generated during the printing process, certain bioprinting mechanics and bioink properties can generate high shear stress on cells. In particular, the stress increases with higher viscosity bioinks, smaller nozzle diameters, and faster speed of deposition in extrusion-based bioprinting.^247,250^

Increasing evidence has shown that cells experiencing lower shear stress when deposited through the nozzle had higher survival rate, and cells that experience higher shear stress often have reduced cell viability and metabolic activity.^169,251,252^ This is also believed to be detrimental to cell’s fate and regeneration capacity.^253^ Therefore, selecting a bioprinting approach that effectively complements the chosen bioink is essential for achieving optimal printability and cell viability throughout the process. It should be noted that an ideal bioink should allow for a more rapid, stable, and cell-friendly bioprinting process with high resolution. For this reason, a nontoxic and modifiable solidification strategies such as chemical cross-linking would also aids in higher cell viability.^52^ Printing parameters, including nozzle diameter, printing speed and extrusion pressure, can also influence overall fabrication time and may contribute to cytotoxicity.^25,254,255^

Lastly, the bioink should provide the bioprinted constructs with in vivo-like mechanical stimulations. It has been highlighted that the cell fate is greatly influenced by hydrogel’s microarchitecture and mechanical strength such as stiffness, porosity, viscoelastic properties and shear stress applied during the bioprinting process.^135,256^ It has been demonstrated that myoblasts detect and respond to the mechanical stiffness of their surroundings, where lower matrix stiffness (~12 kPa) is preferred for the optimal maturation of myotube in 2D.^257,258^

However, the influence of stiffness can have a different effect in 3D microenvironment as myoblasts were encapsulated within hydrogels.^72,137,258,259^ Hajiabbas et al.^179^ examined the effect of different stiffness levels on 3D muscle scaffolds, specifically low (<15 kPa), intermediate (22 ± 1 kPa) and high (100 ± 8 kPa) stiffness. Full expansion of muscle cells in 3D was reported after 2 weeks only in constructs with intermediate stiffness, which also demonstrated the best proliferation compared to other conditions. Morphological observations also revealed that muscle cells in hydrogels with lower and intermediate stiffness were more spindle-shaped, while the myoblasts in the stiffest hydrogels were spherical-shaped. In fact, the stiffness and elasticity of the hydrogels are important factors controlling the cell-matrix interactions, which can direct cell differentiation into various tissue types.

For instance, it is possible to differentiate mesenchymal stem cells (MSCs) towards adipogenic lineages in chitosan-agarose hydrogel matrices due to its relatively low stiffness; whereas when bioprinting using stiffer collagen-based hydrogels, MSCs are mainly differentiated into osteogenic cells.^260^ Additionally, electrical stimulation can be incorporated to promote myotube alignment and differentiation.^261^ Therefore, creating a microenvironment that aligns with the tissue-specific mechanical requirements can promote the spreading and elongation of cells, and governs the effective cell-cell communication, which in turn facilitates long-term cell viability, proliferation, differentiation, and matrix remodelling.

Other mechanical characteristics of bioinks including porosity, swelling and degradation properties should also be considered. Low swelling properties generally lead to limited nutrient diffusion and mechanical stability, which influences long term cell viability and function. The scaffold porosity is therefore important to improve the oxygenation of the bioprinted construct, as well as aiding in the waste removal and nutrient transport.^167^ Biodegradability, whether through hydrolysis, enzymatic activity or oxidation, influences the longevity of a scaffold and how quickly it integrates with host tissues. Thus, controllable biodegradability is preferable as it allows for gradual release of bioactive molecules at tailored degradation rate, also ensuring balanced mechanical strength in vivo.^262^ On a larger scale, the bulk compression modulus of the construct is crucial for maintaining scaffold integrity and under forces. It has been suggested that myoblast cultures typically require a compression modulus of at least 200 kPa to withstand cell-induced deformation during the differentiation process.^69,263^ This could be achieved by combining multiple biomaterials to create regional difference in mechanical properties at the micro-scale. For example, reinforcing frameworks are often used in skeletal muscle tissue engineering to increase the overall compression modulus.

In summary, it is important to tailor the bioink formulation for the optimal compatibility with different types of cells, tissue and bioprinter, regarding their biological, chemical, and mechanical characteristics.^52,125,253,264^ Biomaterials for muscle tissue bioprinting should offer in vivo-like conditions for cells to adhere and migrate, thereby facilitating cell survival, cell proliferation and natural muscle remodelling. This could enhance transplantation outcomes by promoting local muscle regeneration and integration.^24^

## Current challenges and future directions

3D modelling of muscle spindles is an emerging field with significant potential for advancing regenerative medicine and neuromuscular research. Investigation into intrafusal fibre development in vitro remains an under-explored area and requires further understanding, especially in terms of their sensory integration and contributions to neuroplasticity in response to injury or disease. Previous strategies of intrafusal fibre modelling are often not aligned to the native spindle tissue and generally resulted in randomly oriented myofibers, limiting the biological of functional efficiency of the model. 3D bioprinting technology is therefore advantageous as it enables the presence of more complexed internal architecture and biochemical cues, as well as a highly controllable fabrication process. However, several challenges must be addressed to fully realise its capabilities.

The selection of appropriate biomaterials and bioprinting techniques remains a critical challenge in developing physiologically relevant muscle spindle models. Achieving biocompatible and biomimetic scaffolds demands optimisation of material properties and printing methods. To enhance cell viability and intrafusal fibre differentiation, strategies such as fine-tuning shear-thinning properties, controlled and timely growth factor release and the use of support baths during printing have demonstrated significant promise.^97,265^

Bioprinting larger-scale constructs also tend to result in decreased cell viability due to prolonged printing time and limited access to nutrients and oxygen. Addressing these issues requires enhancing scaffold porosity and integrating microvascular networks for improved perfusion. Dividing the printing process into smaller compartments and introducing cells into the bioink immediately before printing each section can also reduce the time cells spend outside their culture media, preserving viability and functionality. For implantation purposes, muscle cells necessitate a robust framework capable of guiding myotube alignment while enduring physiological pressures. Hydrogels should provide sufficient mechanical strength to protect cells while being handleable for surgical manipulation. An ideal construct should allow for easy suturing and customisation to fit patient-specific conditions, whether produced as modular units or large-scale tissues.

Lastly, future studies should explore the integration of proprioceptive sensory innervations and parallel extrafusal fibres into 3D bioprinted muscle spindle constructs. This would enhance physiological relevance and enable better in vitro characterisation of the stretch reflex arc and its functional integration. Such integrated model would allow deeper investigation of cell-cell interactions and the cooperative roles of different cell types in achieving overall spindle functionality.

3D bioprinting holds transformative potential for creating biomimetic muscle spindle models. Beyond applications in regenerative medicine, these models could revolutionise the study of neuromuscular and musculoskeletal disease mechanisms, enabling high-throughput drug screening and the development of personalised therapies. By bridging the gap between in vitro models and in vivo functionality, bioprinted constructs represent a significant leap forward in both clinical and research domains.
